# Foetal microchimerism occurs in pigs

**DOI:** 10.1111/cpr.13333

**Published:** 2022-09-08

**Authors:** Zhengzhu Wang, Jilong Ren, Zhiqiang Han, Jing Wang, Shigang Gu, Yongfeng Zhou, Zhen Han, Yanhui Zhai, Sheng Zhang, Xinglan An, Dawei Yu, Zheng Hu, Tang Hai, Ziyi Li

**Affiliations:** ^1^ Key Laboratory of Organ Regeneration and Transplantation of Ministry of Education, First Hospital Jilin University Changchun China; ^2^ State Key Laboratory of Stem Cell and Reproductive Biology, Institute of Zoology Chinese Academy of Sciences Beijing China; ^3^ Institute of Animal Sciences (IAS) Chinese Academy of Agricultural Sciences (CAAS) Beijing China; ^4^ College of Traditional Chinese Medicine Jilin Agricultural University Changchun China

To the Editor,

The presence of a tiny population of foreign cells or DNA in an organism is known as microchimerism. Microchimerism can occur as a result of iatrogenic operations such as transplantation or transfusion, as well as spontaneously between twins or a mother and foetus.^1^ This results in the presence of maternal cells in the foetal circulation, a condition known as maternal microchimerism (MMc), as well as foetal cells in the maternal circulation, a condition known as foetal microchimerism (FMc).^2^

In 1893, Georg Schmorl initially reported FMc, when he discovered placental trophoblast cells in women who died of eclampsia.^3^ Foetal cells are now well proven to enter the maternal bloodstream throughout both human and rodent gestations.^4^, ^5^ Foetal cells can be detected in multiple organs of the mother, including bone marrow,^6^ kidney,^7^ liver,^8^ heart^9^ and brain.^10^, ^11^ Foetal cells have multilineage potential and can differentiate into different cell types in maternal organs such as blood,^12^ skin^13^ and the central nervous system.^14^ The purpose of foetal cells in maternal tissues is unclear, while numerous ideas include cancer encouragement,^15^ immune surveillance protection^16^ and tissue repair participation,^17^, ^18^ which may be governed by the phenotype acquired by these cells. In addition to humans and mice, FMc has been reported in several animals, such as cattle^19^ and goats.^20^ Cell transfer in multiparous, multichorionic animals like domestic pigs, on the other hand, has received less attention. A study concluded that the epitheliochorial structure of the pig placenta efficiently limits cellular interchange during pregnancy.^21^ Therefore, whether there is microchimerism in domestic pigs still needs to be further studied.

Foetuses with GFP markers would be a better tool for studying foetal microchimerism.^18^, ^22^ In this study, we first created a GFP overexpression vector. The schematic diagram of the GFP overexpression vector construction is shown in Figure 1A. In order to achieve targeted insertion of the expression vector by CRISPR/Cas9 cleavage, a GFP expression vector with a CAG promoter was created and 800 bp left and right homologous arms were added to both sides of the expression vector. We designed identification primers on the outer and inner homologous arms of the insertion gene locus, respectively, to confirm the insertion at the pig Rosa26 locus. HR‐L represented upstream identification and HR‐R represented downstream insertion site identification (Figure 1B). The monoclonal cell line we screened for inserted the target GFP sequence was at the locus of fixation. To further confirm the correct insertion site sequence, PCR products were sequenced, and the results showed that the target insertion sequence of the cell line we obtained was correct (Figure 1C and Method S1). The correctly targeted clones were identified by GFP expression, and used as nuclear donors to construct cloned embryos by SCNT (Figure 1D and Method S2). Compared with the control group, the cloned embryos did not differ significantly in the rate of development to blastocyst and expressed a high level of GFP. The GFP‐1 cell line was utilized to create 1340 cloned embryos, which were subsequently transferred into five surrogate sows. After 30 days, three surrogates detected pregnancy by B‐scan ultrasonography. Finally, 13 piglets were born naturally from three surrogates (Figure 1E and Method S3). The newborn piglets were kept in a clean conventional housing environment. GFP piglets were identified as having green eyes under sunlight and a green whole body under UV light, which was significantly different from WT pigs (Figure 1F).

**FIGURE 1:**
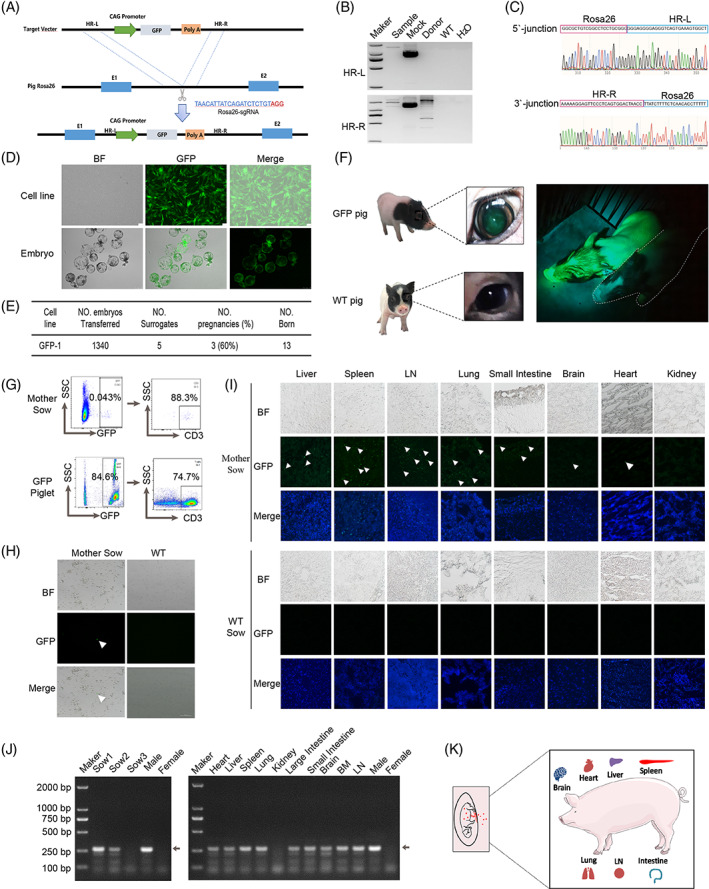
Pig foetal cells across the placental barrier enter the mother during pregnancy. (A) Schematic diagram of the GFP expression gene inserted into the pig Rosa26 locus. We designed a GFP expression vector initiated by the CAG promoter and added 800 bp left and right homologous arms to both sides of the expression vector to achieve targeted insertion of the expression vector by CRISPR/Cas9 cleavage. (B) PCR identification of the fixed‐point insertion. HR‐L represented upstream identification and HR‐R represented downstream insertion site identification. We screened the monoclonal cell line with the target GFP sequence inserted at the fixed‐point position. (C) Sequencing of the PCR products of the identified primers showed that we obtained the correct sequence of the target insertion in the cell line. (D) GFP expression in correctly targeted cells. Scale bars, 1 μm. GFP expression in cloned blastocysts from targeted cells. Scale bars, 100 μm. (E) Summary of SCNT data. (F) GFP piglets were identified as having green eyes under sunlight and a green whole body under UV light, which was significantly different from WT pigs. The piglets were around 3 months old, The dotted part refers to WT pigs. (G) Flow cytometric measurement of porcine leucocytes for GFP cells. PBMCs from cloned GFP piglets contained a certain amounts of GFP positive leukocytes. Leukocytes of the mother sow were also shown a few GFP positive cells, and the second column showed that most of these GFP positive cells were CD3^+^ cells. (H) GFP positive cells were observed under a fluorescent microscope. Leukocytes of the mother sow were shown under specific fluorescence excitation of GFP and brightfield illumination. Arrows pointed to GFP positive leukocytes. Scale bars, 100 μm. (I) Observation of GFP positive cells in solid organs. Cryosections showed the liver, spleen, lymph node, lung, small intestine, brain, heart and kidney of a mother sow and wild type (WT) sow under specific excitation of GFP and brightfield conditions. Arrows point to GFP positive cells. Scale bars, 100 μm. (J) The *SRY* gene was tested in the blood of pregnant sows and in the solid organs of a sow. The arrow showed the location of the *SRY* gene. (K) Pattern diagram of FMc in pigs. During pregnancy, foetal cells entered the mother sow through the placental barrier, and the cells were distributed in different parenchymal organs, including the heart, liver, spleen, lung, LN, intestine and brain. BF, brightfield; BM, bone marrow; LN, lymph node.

To determine whether piglet cells could enter the sows during pregnancy, the peripheral blood of pregnant sows was first examined. After lysis of the red blood cells, the peripheral blood mononuclear cells (PBMC) were used for testing by flow cytometry (Method S4). We found that 0.043% of GFP positive cells were present in PBMC and 88.3% of these cells were CD3^+^ T cells (Figure 1G). Under fluorescent microscopy, we also observed the presence of GFP positive cells in the sow's blood (Figure 1H). To find out whether foetal cells could also circulate through the blood to other organs of the surrogates, we euthanized the sows after delivery and examined the presence of GFP positive cells in other organs by freezing sections (Method S5). More GFP positive cells were found in immune organs such as the spleen and lymph nodes, and small numbers of GFP positive cells were observed in the lungs, liver, intestine, brain and heart, no obvious presence of GFP positive cells was observed in kidney tissues (Figure 1I). Since the genetic material of these cloned GFP piglets was derived from male foetal fibroblasts and the *SRY* gene was only expressed in boars, any foetal cells that entered the mother can be identified by *SRY* detection. By testing the peripheral blood of the surrogate sows, we detected the *SRY* gene in two out of the three surrogate sows (Figure 1J and Method S6). In addition to the kidneys, we detected *SRY* genes in the heart, liver, spleen, lungs, large intestine, small intestine, brain, and bone marrow of the surrogate sows (Figure 1J).

In summary, we found that foetal cells, mostly T cells, can pass the placental barrier and enter the mother's body in pigs. In addition to being present in the blood, these foetal cells were also distributed in the heart, liver, spleen, lungs, lymph nodes, intestines and brain of the sows (Figure 1K). To our knowledge, this is the first study in which FMc has been observed in recipient sows. We discovered foetal microchimera in 67.8% of pregnant sows, suggesting pigs, as well as mice, can be used to examine the incidence and progression of this condition. Our results also showed that pig foetal cells can transfer between the mother and the foetus, indicating that pigs can be used as a large animal model to study maternal‐foetal microchimerism. Unlike mice, pigs have a longer lifetime and can be utilized to study the function of foetal cells in the mother. Taken together, the present findings reveal that pig foetal cells can pass the placental barrier and enter the sow during pregnancy. Of course, further studies are required to determine the type and function of foetal cells in the mother's body.

## AUTHOR CONTRIBUTIONS

Zhengzhu Wang and Jilong Ren performed the experiments and wrote the manuscript. Zhiqiang Han, Jing Wang, Shigang Gu, Zheng Han, Yongfeng Zhou, Yanhui Zhai, Sheng Zhang, Xinglan An and Dawei Yu contributed to the generation of cloned GFP piglets. Zheng Hu and Tang Hai participated in project guidance and revised the manuscript. Ziyi Li conceived the project, supervised the experiments and revised the manuscript.

## CONFLICT OF INTEREST

The authors declare that there are no conflicts of interest.

## Supporting information


**Appendix S1** Supporting Information

## Data Availability

The data that support the findings of this study are available from the corresponding author upon reasonable request.
