# Supplementary material for: Foetal microchimerism occurs in pigs
Source: Cell Prolif. 2022 Sep 8;55(11):e13333. doi: 10.1111/cpr.13333 (PMC9628235; doi:10.1111/cpr.13333)
Supplement: Supplementary file 1 — Appendix S1 Supporting Information [file CPR-55-e13333-s001.docx]

# Supplementary Materials and methods

## Materials: chemicals and animals

Unless otherwise noted, all chemicals and reagents were purchased from Sigma Aldrich Corporation (St. Louis, MO, USA). The Animal Welfare Research Ethics Committee at Jilin University authorized the experimental procedures and standards used to treat and operate on the animals (Approval ID: 20151008-1). Isoflurane was used to establish anesthesia, and every effort was made to make the surgery as painless as possible.

## Method 1: Preparation of GFP fibroblasts and cell line screening

Porcine foetal fibroblasts (pFFs) were isolated from a 35-day-old Bama miniature pig male fetus and cultured in DMEM supplemented with 15% foetal bovine serum. The fibroblasts were electroporated with the Nucleofector 4D Device with a kit following the manufacturer’s instructions (Lonza, Basel, Switzerland). The cell/plasmid mixture was transferred into a cuvette and program DT130 was used for nucleofection, which contained 4 μg of GFP-HDR plasmid, 5 μg of CRISPR/Cas9 plasmid and 3 μg of sgRNA for knock-in cell line screen. We identified the correct insertion of the target GFP sequence into the fixed-point position by PCR technique, in short, the primers were designed on the outer and inner side of the upstream and downstream homologous arms respectively, the primer sequences were D-Rosa26-GFP-UP-KI-F1: CCTGTCAGTTACAGCCTCGG D-Rosa26-GFP-UP-KI-R1: GGCGGGCCATTTACCGT, product fragment length is 963bp, annealing temperature is 63°C; D-Rosa26-GFP-Down-KI-F1: GAGCAAAGACCCCAACGAGA D-Rosa26-GFP-Down-KI-R1: CATTCCAGAGGGAACCACCC, the product fragment length is 1033bp, annealing temperature is 60°C. The correct insertion of the monoclonal cell line was identified by PCR, and then the PCR product was sequenced by sanger to determine that the cell line inserted the correct target sequence.

## Method 2: oocyte collection and in vitro maturation (IVM) and SCNT

Oocyte collection, IVM and SCNT have previously been discussed.^1^ In brief, pig ovaries were obtained from slaughterhouses, stored in 0.9% sodium chloride supplemented with 200 IU/mL penicillin and streptomycin at 35-37 °C, and transferred to the laboratory within 2 hours. A 20-gauge needle linked to a 10 mL syringe was used to aspirate cumulus-oocyte complexes (COCs) from ovarian follicles 3-6 mm in diameter. COCs with at least three layers of cumulus cells were selected and cultured in IVM medium after washing twice with the PBS supplemented with 1% FBS. Fifteen COCs were cultured in a 100 µl drop of maturation medium (TCM-199 supplemented with 26.00 mM sodium bicarbonate, 3.05 mM glucose, 0.91 mM sodium pyruvate, 10.00 µg/ml epidermal growth factor, 50.00 µg/ml luteinizing hormone, 50.00 µg/ml follicle-stimulating hormone, 0.1% polyvinyl alcohol (PVA) [w/v], 0.03% bovine serum albumin (BSA) [w/v] , and 0.1% penicillin/streptomycin (Gibco, MA, USA)) for 22-24 h at conditions of 38.5 °C, 5% CO_2_, 95% air and then transferred to the new drops of the same medium without hormone until 42 h. After maturation, COCs with expanded cumulus cells were treated with 0.2% (w/v) hyaluronidase to remove the cumulus cells via a finely drawn glass capillary pipette. Only oocytes with a visible first polar body (PB1), regular morphology and homogenous cytoplasm were used for SCNT.

Then, mature oocytes and pFFs were placed in manipulation medium containing 7.50 mg/mL cytochalasin B. Under an inverted microscope, the nuclei of mature oocytes and the adjacent PB1 were removed, and the nuclei of GFP bearing fibroblasts were transferred under the zona pellucida of enucleated oocytes. After being cultured in porcine zygote medium 3 (PZM-3, 108.00 mM NaCl, 10.00 mM KCl, 0.35 mM KH2PO4, 0.40 mM MgSO4·7H2O, 25.07 mM NaHCO3, 0.20 mM Na-pyruvate, 2.00 mM Ca-(lactate)2·5H2O, 1.00 mM L-Glutamine, 5.00 mM Basal Medium Eagle amino acids, 10.00 mM Minimum Essential Medium nonessential amino acids, 0.05 mg/mL Gentamicin and 3.00 mg/mL fatty acid-free BSA) for 1 h, the reconstructed/cloned embryos were fused and activated in the fusion medium (0.28 M mannitol, 0.10 mM magnesium sulfate, and 1.00 mM calcium chloride) using an electro-fusion instrument (ECM2001, BTX). The activation parameter was set as two serial DC pulses at 1.5 kV/cm for 100 μs. The cloned embryos were then cultured in PZM-3 medium at 38.5°C, 5% CO_2_ and 100% humidity.

## Method 3: embryo transfer, pregnancy diagnosis and piglets production

After two stable estrus cycles were observed, Bama miniture pigs weighing about 40 kg were selected as embryo transfer recipients. Only surrogate gilts exhibiting a consistent standing reaction were chosen as recipients. The pigs were anesthetized by intravenous injection of propofol (0.1 mg/kg) and then maintained with 15% isoflurane via the nose. After the pigs had no reflexes, surgery was performed for embryo transfer. Cloned embryos cultured for 12 h to 16 h after activation were surgically transferred to the oviduct of estrus recipients. The pregnancy was determined by ultrasonography diagnosis (Philips, CX50) on day 30 after embryo transfer. The cloned piglets were born naturally on the day of their birth.

## Method 4: flow cytometry analysis

Blood was drawn from recipient sows and age-matched control pigs, which was heparin-anticoagulated. RBCs were removed using lysis buffer (555899, BD). The PBMC was chosen as the analysis gate. We employed mouse anti-pig CD3E (LS-C21554, LSBio) to detect CD3^+^ cells. The 1*10^6^ cells were treated for 30 minutes at 4 ̊C with the specified Abs before being rinsed twice with PBS. The flow cytometer (ACSCalibur, BD) was used to evaluate the samples, and the data was processed using the FlowJo software (BD).

## Method 5: fluorescence Histology

Tissue samples (5 mm^3^) were snap frozen in liquid nitrogen before being fixed for 12–24 hours in 4% paraformaldehyde. The samples were washed in PBS, then immersed in 30% sucrose for 24 hours, then frozen in OCT Embedding Compound, and 5 µm slices were made in a frozen slicer and dried on glass slides. 0.3 g/ml of DAPI was used to counterstain the nuclei. After that, the slides were examined under a fluorescence microscope (Nikon).

## Method 6: PCR-based sex identification

Pigs’ genomic DNA was taken from several organs. The NanoDrop One (Thermo Scientific) instrument was used to determine the DNA concentration. Touchdown polymerase chain reaction (PCR) was used for sex determination, with particular primers surrounding a 264 bp section of the SRY gene (SRY-F: 5′- AGGGAGAGAGGGCACAGAAT -3′ and SRY-R: 5′- CACGGTGAAAAGGCAAGTCG-3′). For PCR amplification, 100 ng DNA, 0.5 µM forward and reverse primers, and 10 µl rTaq primer mix (RR901A, TaKaRa) were used for PCR amplification in a total volume of 20 µL. The following were the cycling conditions: an initial denaturation stage at 95°C for 5 minutes was followed by 20 cycles at 95°C for 30 s, 65°C (0.5% decrease per cycle) for 30 s, 72°C for 30 s, followed by another 35 cycles at 95°C for 20 s, 55°C for 30 s, 72°C for 30 s, and a final extension step at 72°C for 10 minutes. Electrophoresis of 2% agarose gel was conducted under conventional conditions of 120 V, 400 mA, and 35 minutes.

## Reference

1. Zhai Y, Zhang M, An X, et al. TRIM28 maintains genome imprints and regulates development of porcine SCNT embryos. *Reproduction*. 2021;161(4):411–424.
